# Colostrum-Derived Exosomal Lactoferrin Promotes Skin Fibroblast Regeneration by Suppressing Inflammatory Responses

**DOI:** 10.3390/cimb47070549

**Published:** 2025-07-15

**Authors:** Chu-Hsun Cheng, Wei-Jer Hong, Chien-Nien Li, Yung-Hsueh Huang, Jeng-Haw Tsai, Chih-Yuan Huang, Jen-Chin Wu, Chan-Yen Kuo, Wen-Chun Kuo

**Affiliations:** 1 Bio-METS Biotech Consulting Co., Ltd., New Taipei City 23544, Taiwan; sephirothx612@gmail.com; 2 JFM Dermatology Clinic, Taipei 104, Taiwan; cafebartender@gmail.com (W.-J.H.); cancervanlee@gmail.com (C.-N.L.); 3 20 SKIN Clinic, Taichung City 404, Taiwan; 20muteki@gmail.com; 4 Beauty Humanity Clinic, New Taipei City 234, Taiwan; jenghaw@cplan.com.tw; 5 Buddhist Tzu Chi General Hospital, Taichung Branch, Taichung City 427213, Taiwan; huangpcy@gmail.com; 6 Mclinic Clinic, Taichung City 408, Taiwan; 7 Wu Jenchin Dermatology Clinic, Pingtung City 813755, Taiwan; 0939776239@gmail.com; 8 Institute of Oral Medicine and Materials, College of Medicine, Tzu Chi University, Hualien 970, Taiwan

**Keywords:** exosomes, lactoferrin, colostrum, inflammation, fibroblasts, wound healing, cytokines, skin regeneration

## Abstract

Lactoferrin (LF), a multifunctional glycoprotein found abundantly in bovine colostrum, is known for its regenerative and anti-inflammatory properties. In this study, we investigated the wound healing and immunomodulatory effects of colostrum-derived exosome-encapsulated lactoferrin (EV-exoLF) on dermal fibroblasts. EV-exoLF was isolated and characterized via nanoparticle tracking analysis and flow cytometry. Functional assays demonstrated that EV-exoLF significantly promoted fibroblast viability and migration in both mouse NIH/3T3 and human HS-68 cell lines. Furthermore, EV-exoLF reduced the expression of pro-inflammatory cytokines (IL-1 and IL-6) and phosphorylated JNK in lipopolysaccharide (LPS)-treated fibroblasts. These findings suggest that EV-exoLF not only enhances fibroblast-mediated wound closure but also mitigates inflammation, highlighting its therapeutic potential in skin regeneration. Colostrum-derived exosomal lactoferrin may serve as a promising natural, cell-free strategy for managing inflammatory skin conditions and improving wound healing outcomes.

## 1. Introduction

The skin is a complex organ composed of the epidermis, dermis, subcutaneous fat or dermal adipocyte layer, and skin appendages, including hair follicles, sweat glands, and sebaceous glands. The main functions of the skin are to provide a barrier to protect an organism from deleterious environmental impacts (thermal, chemical, physical, mechanical, and chemical injury, as well as microbiological substances) and to maintain hemostasis in the body [1,2]. The skin serves as a protective shield from the external environment and is constantly exposed to potential harm; hence, wound healing is important for the survival of all higher organisms [3,4].

Dermal wound healing is a complex and dynamic process that occurs in living organisms and requires the coordination of multiple types of cells, tissues, growth factors, and the extracellular matrix (ECM). Wound healing is a vital process among species and can be spatially and temporally grouped into hemostasis, inflammation, cellular proliferation, and tissue remodeling [5,6,7,8]. Although these phases are mutually exclusive, they can overlap in time. However, the outcomes of skin wound healing differ between species. Some lower vertebrates, such as fish (zebrafish) and amphibians (newts), have a remarkable ability to regenerate the epidermis, dermis, and scales in a scarless manner [9,10]. Contrastingly, in adult mammals, including humans, scar formation is a formidable challenge when aiming to achieve such regeneration. Scar formation is a normal and unavoidable outcome of the tissue remodeling phase that can act as a requirement for the fundamental function of the skin in preventing infection and dehydration. However, scar formation can be unfavorable [6,8,11]. The appearance of scar tissue lacking skin appendages (oil glands, sweat glands, hair follicles, etc.) is significantly different from that of the original intact skin. Scars from injuries or burns can have devasting cosmetic and psychological consequences, thereby reducing the quality of life of an individual [12]. Skin appendages are critical for biological and physiological functions of the body. They may act as a large mass of cells for wound healing, promoting wound closure and tissue repair [13]. Furthermore, previous studies have suggested additional roles for hair follicles and sebaceous glands as sensory and thermoregulatory organs [14,15]. Therefore, scar formation prevents the complete recovery of skin function. Although it is impossible to completely prevent scar formation during wound healing, the ability to restore the skin to its original state and methods for preventing or reducing scar formation are the main goals of regenerative medicine.

Wound healing predominantly aims to promote rapid healing and the regeneration of skin tissue with a good appearance. However, wound healing is a dynamic process involving the interaction of multiple factors. Therefore, although the current industry has a considerable understanding of cell and molecular biology, the incidence of hypertrophic scars or keloid formation remains relatively high. Current evidence demonstrated that high levels of inflammation in injured skin directly correlate with excessive dermal scarring and the formation of abnormal pathological scars [5,6,8,12,16]. In the wound site, inflammatory cells are activated that then secrete more inflammatory cytokines to give rise to additional inflammation and the expression of detrimental cytokines, including PDGF, TGF-β, and interleukin (IL)-4/IL-13. IL-4/IL-13 induces the differentiation of fibrocytes into fibroblasts. Dysregulation of inflammation can promote pathological scar formation by increasing ECM deposition and the abundance of over-differentiated cells, such as fibroblasts and keratinocytes, at the wound sites [17,18]. Furthermore, TGF-β acts on fibroblasts and promotes their differentiation into myofibroblasts. These activated cells subsequently generate excessive levels of collagens and other ECM components that play an important role in scar formation. Therefore, the regulation of inflammation during wound healing is critical for effective tissue repair and the prevention of excessive scar formation [19,20,21].

Promoting wound healing with functional recovery of the skin layer and appendages is the primary goal of regenerative medicine. To achieve skin healing and regeneration, various methods to accelerate the wound healing process, such as medications, growth factor delivery, wound dressings, and skin grafts, have been utilized for different types of wounds [22,23,24,25,26]. Advanced biomaterials can be used in the process of wound healing to provide physical support, serve as vehicles to carry healing-promoting factors, promote cell functions, and facilitate the formation of new tissue at the target wound site [27,28,29]. Exosomes derived from living cells show great promise in novel approaches used to improve skin wound healing [30,31,32]. Exosomes are nanosized extracellular vesicles (EVs) with a lipid bilayer membrane that carries nucleic acids, proteins, lipids, and other bioactive substances. Exosomes play a critical role in the dynamic processes of cellular communication and intercellular signaling in health and disease by transferring cellular cargo, such as functional proteins, metabolites, and nucleic acids, to recipient cells. Exosomes can be used as noninvasive biomarkers for the detection and diagnosis of diseases and also act as natural drug delivery vehicles for the treatment of cancer or other diseases [30,33,34]. As exosomes are highly biocompatible and capable of crossing biological barriers to travel into deep tissue, they have emerged as potential functional nanomaterials in regeneration medicine and tissue engineering.

Several studies have shown that milk contains abundant exosomes, and they have thus recently been developed as promising new nanovehicles in biotherapeutics for treating various disease [35,36,37]. Milk is not only a resource of nutrients but also contains hundreds to thousands of distinct bioactive molecules that protect against infection and inflammation and contribute to immune maturation, organ development, and healthy microbial colonization. Moreover, milk exosomes contain specific milk proteins, such as caseins, lactoglobulin, lactoferrin (LF), CD36, and polymeric immunoglobulin receptor precursor [38]. A previous study demonstrated that colostrum exosomes have significantly higher expression levels of LF and kappa-casein [39]. LF is a bilobate iron-binding glycoprotein of the transferrin family that is present in large amounts in colostrum and all mammalian milk [40,41]. In milk, LF plays an important role in promoting antimicrobial effects because of its ability to sequester iron from microorganisms. Furthermore, LF can directly interact with microorganisms to exert antimicrobial activity via an iron-independent pathway [42,43,44,45]. Therefore, milk-derived exosomes are promising therapeutic agents for dermal wound healing.

The present study aimed to investigate the bioactivities of exosome-encapsulated LF (EV-exoLF) in mouse 3T3/NIH embryo fibroblast cells and the human new foreskin fibroblast cell line, HS-68 (CRL-1635). The effects of these exosomes on wound healing were assessed using a scratch assay and in vitro studies of the expression of pro-inflammatory cytokines and phosphorylated c-Jun N-terminal kinase (pJNK). Overall, our study provides a new perspective on this promising cell-free therapeutic strategy for wound healing.

## 2. Materials and Methods

### 2.1. Reagents

Dulbecco’s Modified Eagle’s Medium (DMEM) was obtained from Thermo Fisher Scientific (Waltham, MA, USA). Penicillin/streptomycin was obtained from BioIndustry (London, UK). PhosSTOP and complete ULTRA Tablets were purchased from Roche (Basel, Switzerland). Anti-phospho-JNK (#AP0631) and β-actin (#AC004, actin) antibodies was purchased from Abclonal (Woburn, MA, USA). Lipopolysaccharide (LPS) from *Escherichia coli* O111:B4 was purchased from Sigma-Aldrich (#L4130; St. Louis, MO, USA).

### 2.2. EV-exoLF Extract Isolated from Colostrum

Colostrum from healthy cows was purchased from Morinaga Milk Industry Co., Ltd. (Tokyo, Japan). Milk exosomes were isolated from colostrum via ultracentrifugation as described previously, with a slight modification. All centrifugation steps were performed at 4 °C. Briefly, milk was centrifuged at 5000× *g* for 30 min and then at 12,000× *g* for 1 h to remove milk fat globules, somatic cells, and cell debris. The defatted milk was stored at −80 °C until use. After the milk was thawed, it underwent two brief centrifugation steps to remove debris remaining from the freeze/thaw step: 1200× *g* for 10 min and 300× *g* for 10 min. The supernatant was then passed through a 0.2 μm filter for sterilization to eliminate larger particles, including bacteria. The exosome pellet was obtained via ultracentrifugation at 100,000× *g* for 2 h at 4 °C using an L-90K ultracentrifuge (Beckman Coulter, Brea, CA, USA). Following removal of the exosome-free supernatant, the pellet was resuspended in phosphate-buffered saline (PBS) and stored at −20 °C until use in subsequent experiments.

### 2.3. Cell Line Culture

The mouse 3T3/NIH embryo fibroblast (CRL-1658) and human HS-68 new foreskin fibroblast (CRL-1635) cell lines were purchased from the American Type Culture Collection (Manassas, VA, USA). Fibroblasts were cultured in high-glucose DMEM at 37 °C in a humidified atmosphere containing 5% CO_2_. The medium was supplemented with 10% Newborn Calf Serum and 1% penicillin/streptomycin. The fibroblasts were plated in 100 mm culture dishes at a density of 5 × 10^5^ cells/dish.

For LPS stimulation, the cells were plated on 35 mm dishes and passaged 1:4 (35 mm) using 0.25% trypsin when they reached 80–90% confluence. Then, these cells were randomly divided into three groups: control (n = 3), LPS (400 ng/mL; n = 3), and LPS plus EV-exoLF groups (LPS, 400 ng/mL; EV-exoLF, 200 µg/mL; n = 3).

### 2.4. CCK-8 Assay

NIH/3T3 and HS-68 cells were counted, and approximately 5 × 10^3^ cells were seeded into each well of a 96-well culture plate (Corning Inc., Corning, NY, USA). After incubation at 37 °C in a humidified atmosphere with 5% CO_2_ for 24 h, the cells were washed and treated with various concentrations of EV-exoLF (10, 50, 100, 200, 250, and 300 μg/mL) for 24, 48, and 72 h. An untreated control group was included for comparison. Each condition was tested in five replicates. Following treatment, 10 μL of CCK-8 reagent (MedChemExpress Ltd., Monmouth Junction, NJ, USA) was added to each well and incubated for 2 h at 37 °C. The optical density (OD) was then measured at 450 nm using a multifunction microplate reader (Biochrom EZ Read 400, Biochrom, Cambridge, UK). The results represent the average of three independent experiments and are expressed as mean ± SEM.

### 2.5. Nanoparticle Tracking Analysis Measurements

The size and concentration of exosomes in a given sample were determined using a NanoSight NS300 instrument (Malvern Panalytical, Malvern, UK; Cornell NanoScale Science and Technology Facility, Ithaca, NY, USA) as described previously, with a slight modification [46,47]. Settings were applied according to the manufacturer’s software manual (NanoSight NS300 User Manual, MAN0541-01-EN-00, 2017). Briefly, the EV-exoLF samples were diluted in PBS to a final volume of 1 mL and then transferred to a disposable cuvette. The ideal measurement concentrations were determined by pretesting the ideal particle-per-frame value (20–100 particles/frame). A milliliter of the dilution sample was injected into the sample chamber with sterile syringes, and 60 s videos of each sample were taken and analyzed to determine the concentration and size of the individual EVs under a constant flow rate of 70 µL/min at room temperature. The results obtained were averaged.

### 2.6. Flow Cytometry Analysis of EV-exoLF

To detect the expression levels of surface marker proteins in EV-exoLF, flow cytometry analysis was performed using the PS Capture Exosome Flow Cytometry Kit (FUJIFILM Wako Chemicals, Osaka, Japan), according to the manufacturer’s instructions. Briefly, EV-exoLF samples were incubated with an exosome-binding enhancer and exosome capture beads at room temperature for 1 h. The exosome-binding beads were washed and resuspended in wash buffer for immunostaining. After washing the beads, the exosomes were stained with fluorescence-labeled antibodies (BB700 Mouse Anti-Human CD9 [745827; BD, Franklin Lakes, NJ, USA], FITC mouse anti-human CD63 [55728; BD], and RB780 Mouse Anti-Human CD81) for 60 min at room temperature in the dark. The exosome-binding beads were washed and resuspended in washing buffer and analyzed using a CytoFLEX flow cytometer (Beckman Coulter, Lane Cove West, Australia) with the Kaluza software (V2.1).

### 2.7. Wound Healing Assay

After reaching 90% confluence within 3–5 days, the cells were rinsed in PBS, detached via trypsinization with 0.05% trypsin for 5 min at 37 °C, and centrifuged (1500 rpm). Subsequently, the cells were suspended in culture medium and seeded on a 24-well plate using an Ibidi Culture-Insert 2 Well (Cat. No. IB-80209; ibidi, Fitchburg, WI, USA). The NIH/3T3 and HS-68 cells were seeded at a density of 50,000 and 100,000 cells/50 μL/chamber, respectively. The Ibidi Culture-Insert 2 Well was removed 24 h after seeding, creating a gap for cell migration. The cells were washed three times, and the culture medium replaced with serum-free DMEM in the presence or absence of 0.4% EV-exoLF. Cell morphology was observed at 0, 24, and 48 h to quantify confluency and assess cell migration. Light micrographs were captured with a Leica 090-137-001 inverted microscope attached to a digital camera (EC3; Leica, Wetzlar, Germany) using Leica LAS EZ Imaging software v3.4.

### 2.8. Enzyme-Linked Immunosorbent Assays

HS-68 cells were cultured in a six-well plate and treated with 400 ng/mL LPS for 6 h to increase the expression levels of pro-inflammatory cytokines. After treatment with LPS, the HS-68 cells were treated with 200 µg/mL EV-exoLF for 4 h. Subsequently, cell supernatants were collected. The levels of IL-1 (#orb2812066, Biorbyt, UK) and IL-6 (RayBiotech, Peachtree Corners, GA, USA) were measured in the cell culture supernatants using an enzyme-linked immunosorbent assay (ELISA) reader (Biochrom EZ Read 400 Microplate Reader; Biochrom, Cambridge, UK), according to the manufacturer’s instructions. The dilution of the supernatant was 1:2.

### 2.9. Western Blotting

Sodium dodecyl sulfate-polyacrylamide gel electrophoresis was performed using 10% or 12% acrylamide gels, with equal amounts (30 µg) of protein loaded per lane. After electrophoresis, the proteins were transferred to polyvinylidene fluoride membranes at 350 mA for 2 h. For blocking, the membranes were soaked in 5% nonfat milk at room temperature for 1 h at 75 rpm. The membrane was then incubated with primary antibodies at 4 °C overnight. The following day, the membranes were washed thrice with TBS buffer containing 0.2% Tween 20 (Bionovas, Halifax, NS, Canada) at room temperature for 10 min each. The cells were then incubated with a secondary antibody conjugated to horseradish peroxidase (HRP) at a 1:10,000 dilution for 1 h at room temperature. After washing in TBS buffer with 0.2% Tween 20, Western HRP substrate (Luminata Classico; Millipore, Darmstadt, Germany) was used to develop the fluorescent signal, which was visualized using a ChemiDoc XRS+ System (Bio-Rad Laboratories, Hercules, CA, USA).

## 3. Results

### 3.1. Characterization of EV-exoLF Derived from Colostrum

**Figure 1:**
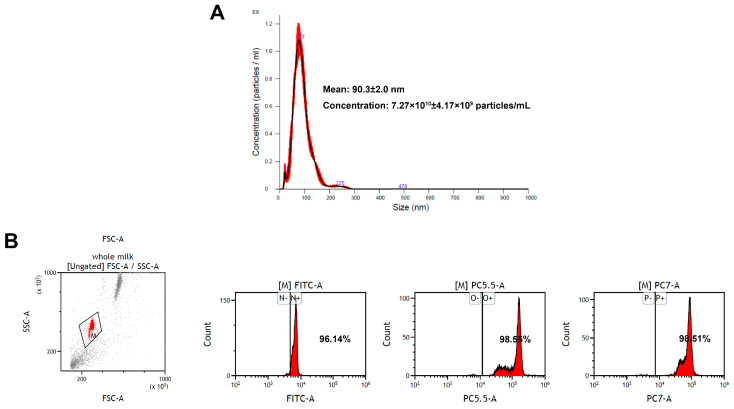
Characterization of exosome-encapsulated lactoferrin (EV-exoLF) derived from colostrum. (**A**) Size detected via nanoparticle tracking analysis. (**B**) Flow cytometry scatter plots of the expression levels of representative EV-exoLF surface biomarkers (CD9, CD63 and CD81).

Exosomes are small EVs with diameters ranging from 30 to 150 nm [33]. In the present study, EV-exoLF was isolated using a modified ultracentrifugation method. Their average size measured using nanoparticle tracking analysis were 90.3 ± 2.0 nm with a concentration of 7.27 × 10^10^ ± 4.17 × 10^9^ particles/mL. Flow cytometry analysis demonstrated the presence of exosomal marker proteins (CD9, CD63, and CD81). Together, these results confirmed the successful isolation of EV-exoLF (Figure 1).

### 3.2. EV-exoLF Exhibits Dose- and Time-Dependent Effects on Cell Viability

**Figure 2:**
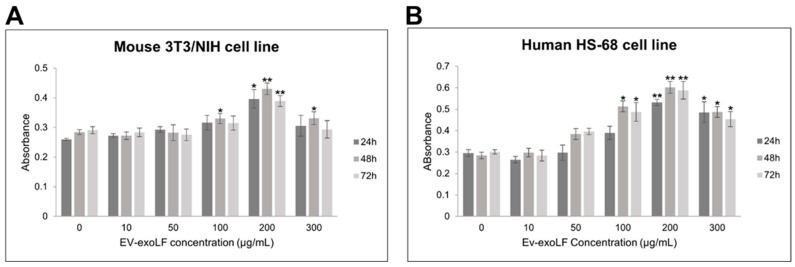
CCK-8 assay results showing the dose-dependent effects of EV-exoLF on the viability of mouse NIH/3T3 (**A**) and human HS-68 (**B**) cell lines following 24, 48, and 72 h of exposure. Untreated cells served as the control group. Data are presented as mean ± SEM from three independent experiments (n = 3). Statistical significance was determined using Student’s *t*-test; *p* < 0.05 (*), *p* < 0.01 (**) versus untreated controls.

To evaluate the potential cytotoxicity of EV-exoLF, cell viability assays were performed on murine NIH/3T3 fibroblasts and human HS-68 fibroblast cells using the CCK-8 assay. Cells were treated with increasing concentrations of EV-exoLF (10, 50, 100, 200, 250, and 300 μg/mL) for 24, 48, and 72 h. As shown in Figure 2, EV-exoLF exerted a dose- and time-dependent effect on both cell lines. At lower concentrations (10–100 μg/mL), cell viability remained comparable to untreated controls across all time points. However, at higher concentrations (200–300 μg/mL), a significant reduction in viability was observed, particularly after 48 and 72 h of treatment (Figure 2). These findings demonstrate that EV-exoLF is well tolerated at lower concentrations but exerts cytotoxic effects at higher doses and prolonged exposure times, providing a basis for selecting non-toxic working concentrations for subsequent functional assays.

### 3.3. Capacity of EV-exoLF to Induce Cell Proliferation During the In Vitro Scratch Assay

**Figure 3:**
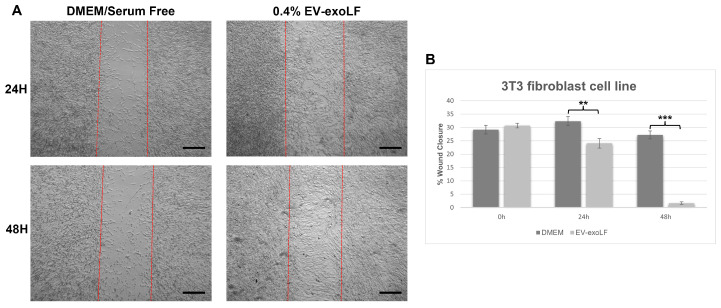
EV-exoLF enhances the rate of wound closure in the mouse NIH/3T3 fibroblast cell line. (**A**) Representative light micrographs of the scratch wound assays used to evaluate migration rate at 24 and 48 h. A significant increase in the extent of wound closure was observed in the EV-exoLF group compared with that in the control Dulbecco’s Modified Eagle’s Medium (DMEM)/serum-free groups at 24 and 48 h. Scale bar: 200 µm. (**B**) Statistical analysis of the wound closure area performed using ImageJ software (1.54p) (n = 3 per group; ** *p* < 0.01, *** *p* < 0.001, one-way ANOVA and Tukey’s post hoc test). Error bars denote the standard error of the mean (SEM).

**Figure 4:**
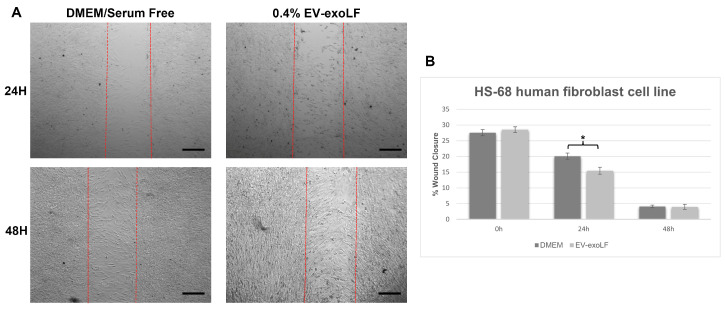
EV-exoLF enhances the rate of wound closure in the human new foreskin fibroblast cell line, HS-68 (CRL-1635). (**A**) Representative light micrographs of the scratch wound assays used to evaluate migration rate at 24 and 48 h. A significant increase in the extent of wound closure was observed in the EV-exoLF group compared with that in the control DMEM/serum-free groups at 24 h, but no significant difference observed between the EV-exoLF and control groups at 48 h. Scale bar: 200 µm. (**B**) Statistical analysis of the wound closure area performed using ImageJ software. (n = 3 per group; * *p* < 0.05, one-way ANOVA and Tukey’s post hoc test). Error bars denote the SEM.

To investigate the effect that exosome stimulation has on cell healing, we cultured mouse NIH/3T3 and human HS-68 fibroblast cell lines and assessed cell migration using a wound scratch assay (Figure 3 and Figure 4). For the migration assay, the NIH/3T3 and HS-68 cell lines were cultured in serum-free DMEM with or without 0.4% EV-exoLF after reaching a density of 90% and creating a scratch, and the percentage of wound closure calculated after 24 and 48 h. For the mouse NIH/3T3 cell line, treatment with 0.4% EV-exoLF significantly enhanced migration to the scratch area after 24 (control: 32.3 ± 5.9%, EV-exoLF: 24.1 ± 7.2%; ** *p* < 0.01) and 48 h (control: 27.2 ± 5.5%, EV-exoLF: 1.6 ± 2.3%; *** *p* < 0.001) compared with that of the control group (Figure 3). Next, we investigated whether EV-exoLF could induce the same effect in human HS-68 cells. We confirmed that EV-exoLF also significantly promoted the scratch closure of human HS-68 cell lines at 24 h (control: 20.1 ± 1.0%, EV-exoLF: 15.5 ± 1.1%; ** *p* < 0.01) compared with that of the control (Figure 4), but no significant difference was observed between the control and EV-exoLF groups at 48 h (control: 4.1 ± 0.4%, EV-exoLF: 4.0 ± 0.8%). These data indicated that EV-exoLF was capable of inducing dermal fibroblast migration and scratch wound closure.

### 3.4. EV-exoLF Inhibited the Inflammatory Response by Modulating Key Factors in Pro-Inflammation

**Figure 5:**
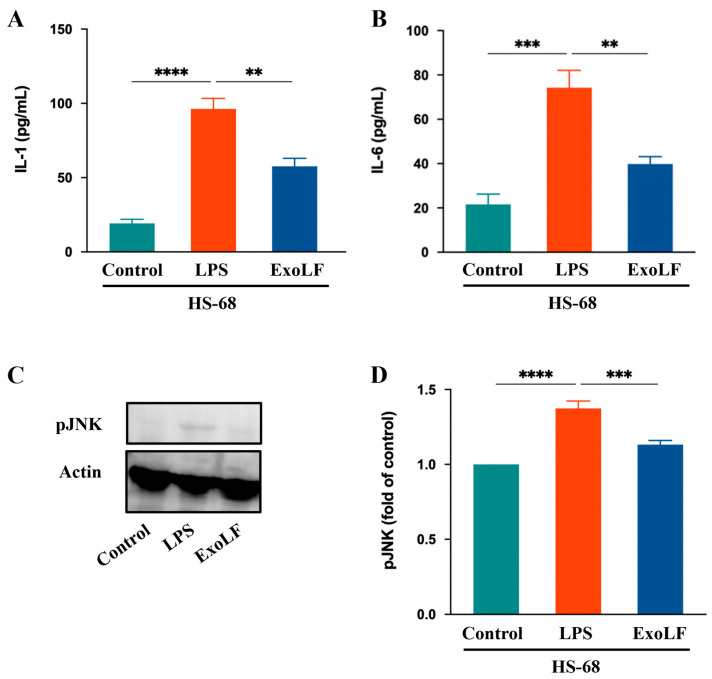
The effect of EV-exoLF on the inflammatory response in the lipopolysaccharide (LPS)-treated HS-68 cell line. (**A**,**B**) Interleukin (IL)-1 and IL-6 results of HS-68 cells conditioned after treatment with or without EV-exoLF. (**C**) Changes in the expression of pJNK. Actin was used as an internal control. (**D**) Quantitative results showing the pJNK protein levels assessed using ImageJ. All data are presented as the mean ± standard deviation (SD) (n = 3. ** *p* < 0.01, *** *p* < 0.001, **** *p* < 0.0001, one-way ANOVA and Tukey’s post hoc test). Error bars denote the SD.

IL-1 and IL-6 are crucial pro-inflammatory cytokines that play central roles in mediating pathological scar formation [48]. To confirm the anti-inflammatory effects that EV-exoLF has on IL-1 and IL-6 production in HS-68 cells, we measured their expression levels using ELISA. The results showed that EV-exoLF significantly reduced the production of both IL-1 and IL-6 in HS-68 cells. In addition, JNK signaling participated in wound healing processes, such as cell proliferation, inflammation, and the immune response. Excessive activation of the JNK pathway can exacerbate inflammatory responses and promote scar formation [49]. In the present study, we investigated whether EV-exoLF could modulate JNK activation in HS-68 cells after LPS treatment. The results showed that EV-exoLF administration markedly downregulated pJNK protein expression in LPS-treated HS-68 cells. Taken together, these findings suggest that EV-exoLF alleviated the inflammatory response through specific pathways (Figure 5).

## 4. Discussion

The skin is the largest organ in the human body and serves multiple functions; therefore, the rapid healing of skin wounds is essential. As regenerative processes occur during wound healing, the migration of fibroblasts and keratinocytes plays a key role in determining the efficiency of the initial wound healing phase [50,51]. In the present study, we showed that EV-exoLFs, milk exosomes isolated from colostrum, were capable of promoting cell migration and tissue regeneration in a wound healing assay and suppressing detrimental immune responses in an LPS-induced inflammation model, justifying the significance and potential of EV-exoLF in treating skin wounds.

Wound healing is a natural process of skin and epidermal tissue regeneration after an injury. It requires the coordination of cell migration, proliferation, ECM deposition, and remodeling, as well as the regulation of inflammation and angiogenesis. Although small skin wounds can heal within a few days, larger injuries caused by trauma, acute illness, or major surgery may take weeks to heal and often result in fibrotic scarring, which can affect tissue function [11,18]. EV-exoLF significantly enhanced fibroblast proliferation and migration, which are key processes required for enhancing wound healing. The increase in cellular proliferation observed at wound edges following EV-exoLF treatment was due to their potential to activate regenerative processes. This suggests that EV-exoLF could serve as an effective therapeutic tool for enhancing wound healing, especially in cases involving reduced inflammation, such as LPS-induced wounds.

Exosomes are EVs released by multiple cell types into the extracellular space under physiological processes, and their secretion is upregulated under pathological conditions. Several studies have implicated exosomes in the progression of various pathologies, including inflammatory and chronic diseases, trauma, cancer, etc. [33,34,52,53]. Therefore, exosomes could be used as precise diagnostic tools to treat a wide range of disorders. Exosomes have been examined for their therapeutic potential in various medical applications, including tissue repair, immune modulation, and cancer treatment. Furthermore, exosomes have shown the potential to play notable roles in cosmetics, skin care, tissue regeneration, and dermatological diseases due to their ability to regulate skin health, immunity, and regeneration. Exosomes contain a wide variety of bioactive components and specific molecules that can promote skin healing, hydration, and protection. These molecules support collagen synthesis, reduce inflammation, and protect the skin from environmental stress. In addition, exosomes can enhance the effectiveness of other active ingredients, such as hyaluronic acid, peptides, and antioxidants [54].

The inflammatory response is an important self-protective mechanism of the body that helps prevent pathogen invasion and plays a crucial role in triggering wound healing. However, if the inflammatory response does not slow or cease at the appropriate time and becomes uncontrolled, excessive inflammation during the wound healing stages can cause abnormal scarring, which in turn may result in the formation of irregular fibrous tissues, such as hypertrophic and keloid scars [55]. The main visible difference between hypertrophic scars and keloids is the extent to which the scar spreads beyond the original wound area. They are characterized by continuous local inflammation and excessive ECM deposition. However, studies have revealed that keloids appear to be a more sustained, stronger inflammatory and aggressive fibrotic disorder than hypertrophic scars are [56,57]. Previous studies have demonstrated that mesenchymal stem cell (MSC)-derived conditioned medium or exosomes have immunomodulatory effects and can reduce the inflammatory response by suppressing immune cells, overactivated fibrotic cells, and the synthesis of inflammatory cytokines [58,59]. Adipose-derived MSC (ADSC)-derived exosomes have been reported to inhibit collagen I (Col1), collagen III (Col3), fibronectin, and α-smooth muscle actin (α-SMA) gene and protein expression in keloid fibroblasts [60]. In addition, ADSC-derived exosomes effectively inhibited the proliferation and migration of hypertrophic scar-derived fibroblasts by decreasing the expression of Col1, Col3, α-SMA, IL-17RA, and p-Smad2/p-Smad3 [61]. Hence, MSC-derived exosomes could serve as a promising therapeutic strategy for attenuating the transdifferentiation of fibroblasts into myofibroblasts and inhibiting the gene and protein expression of ECM-related factors in hypertrophic scars and keloids.

Although MSC-derived exosomes have numerous advantages in pathological scar treatment, the source of cells and standardization of the culture process both affect subsequent therapeutic outcomes. Current cell culture and exosome purification methods limit large-scale exosome manufacturing for clinical use. Typically, the large-scale production of exosomes requires cell conditioned media, which results in high production costs [62]. Unlike cell or cell line culture systems, natural biological fluids, such as exosomes derived from breast milk, are ideal platforms that can be used to achieve large-scale production for cosmetic or clinical use [63]. Bovine milk, particularly colostrum, is one of the most promising platforms and a scalable source of exosomes for mass production. Colostrum-derived exosome are significantly enriched with proteins, such as casein, β-lactoglobulin, α-lactalbumin, LF, and other active molecules, that can potentially regulate the immune response and growth [64]. Several studies have shown that milk and milk-derived exosomes have many health-promoting functions due to their antibacterial, antifungal, and anticancer effects [39,65,66]. In addition, milk-derived exosomes have been confirmed to exert therapeutic effects in preventing and treating intestinal disease [65,66]. Bovine colostrum-derived exosomes have also been reported to repair and rejuvenate all phases of skin damage, representing a new therapeutic option in regenerative medicine [67,68]. A recent study showed that bovine-derived milk exosomes exerted antioxidant and anti-inflammatory effects and modulated the gene and protein expression of TGFβ3 and TGFβ1 in scar-free wound healing [69]. Exosomes derived from bovine milk also demonstrated the potential to repair the induction of UV-irradiated skin aging and damage by suppressing intracellular reactive oxygen species in epidermal keratinocytes and matrix metalloproteinase expression in dermal fibroblasts [67]. Our data showed that colostrum-derived exosomes containing LF can significantly facilitate the migration and wound closure of fibroblasts. Fibroblast migration and proliferation are essential components of the wound healing process. We suggested that the colostrum-derived exosomes, EV-exoLFs, are able to exert this effect due to the high presence of activated factors, which promote the functions of fibroblasts.

Exosomes were reported to accelerate wound healing by attenuating inflammation, inhibiting oxidative stress damage, and regulating cellular function, ECM production, and remodeling. The results of our study demonstrated that LPS-induced overexpression of the pro-inflammatory cytokines, IL-1, IL-6, and pJNK, could be downregulated by EV-exoLFs. Previous studies have demonstrated that IL-1, IL-6, and JNK play important roles in promoting scar formation [48,70,71]. IL-1, which is subdivided into IL-1α and IL-1β, is an important pro-inflammatory cytokine not only for inflammation but also healing and downstream scar formation, and it is mainly produced by macrophages during tissue remodeling. During the inflammatory phase, IL-1 has a pronounced influence on skin homeostasis and wound repair and is highly produced by keratinocytes. However, high levels of IL-1 are related to negative effects in the process of skin inflammation and promote the synthesis and regulation of other inflammatory mediators [72,73]. Pleiotropic IL-6 initiates the healing process and regulates the cell network composed of fibroblasts, lymphocytes, macrophages, keratinocytes, and vascular endothelial cells. IL-6 also promotes tissue remodeling and cell proliferation. Nonetheless, its overexpression may play a key role in prolonging the inflammatory phase during wound healing, which increases the incidence of scar formation [16,70]. Wound healing progresses through the phases of hemostasis, inflammation, proliferation, and remodeling and is accompanied by re-epithelialization, which restores dermal function. JNK, a member of the mitogen-activated protein kinase family, is a cell signaling molecule expressed in most cell types and is associated with the regulation of several cellular processes involved in wound healing and scar formation [49,74,75]. Previous studies have demonstrated that ECM production by fibroblasts is associated with JNK signal activation. JNK inhibition was found to significantly reduce the transition of dermal fibroblasts to the myofibroblast phenotype and collagen deposition [76]. In this study, ELISA for IL-1 and IL-6 secretion and Western blot analysis for phosphorylated JNK (p-JNK) were performed exclusively in HS-68 human fibroblast cells. This decision was based on the objective to evaluate the anti-inflammatory effects of EV-exoLF in a human-relevant in vitro model, given the translational importance of human cytokine response patterns. Although NIH/3T3 mouse fibroblasts were included in the viability assays to assess general cytocompatibility, cytokine secretion and signaling pathway activation can differ significantly across species. Therefore, to ensure physiological relevance and consistency in the inflammatory readouts, we limited downstream mechanistic assays to the HS-68 cell line. Future studies will aim to extend these findings to murine models to further explore species-specific responses.

Furthermore, LF is abundant in milk exosomes [39] and may play a pivotal role in modulating the LPS-induced overexpression of pro-inflammatory cytokines. LF exerts numerous biological effects on the wound healing process, including the regulation of cell proliferation and differentiation and the promotion of antimicrobial and anti-inflammatory effects. Studies have shown that during wound healing, LF promotes fibroblast proliferation and migration [77], as well as the secretion of collagen and hyaluronic acid. In contrast, collagen can enhance keratinocyte maturation and proliferation and contribute to the healing of damaged tissues [78]. Our findings are consistent with those of previous results showing that LF promotes fibroblast migration in an in vitro scratch wound assay. During bacterial infections, the bacterial endotoxin, LPS, triggers an inflammatory response. LF can bind to LPS to block its interaction with cell membrane receptors, thereby reducing the production of pro-inflammatory cytokines, such as tumor necrosis factor-α, IL-1, and IL-6, and enhance the secretion of the anti-inflammatory cytokine, IL-10, to regulate the immune response [79]. However, pro-inflammatory factors produced by macrophages or dendritic cells stimulate neutrophils to secrete large amounts of LF during injury or pathogen infection. LF can bind to pathogens or immune cells, such as macrophages, suppress the production of inflammatory cytokines, terminate the inflammatory response, and promote advancement to the tissue repair phase [80]. Our findings showed a significant reduction in protein expression levels for IL-1, IL-6, and pJNK in LPS-stimulated fibroblast cell lines upon treatment with EV-exoLF. These results are consistent with the findings of a previous study showing that LF downregulates the expression of pro-inflammatory cytokines induced by LPS. However, the specific mechanisms through which milk exosomes modulate LPS-induced inflammation require further investigation.

Although the reduction in phosphorylated JNK (p-JNK) levels observed in this study suggests that EV-exoLF may suppress JNK-mediated inflammatory signaling, it remains unclear whether this pathway is directly involved in the observed enhancement of wound healing. The JNK signaling pathway is known to regulate not only inflammation but also cell migration and proliferation in various contexts [81]. While our data show that EV-exoLF treatment promotes fibroblast migration and downregulates p-JNK expression, we acknowledge that this represents a correlative observation. Further studies using JNK-specific inhibitors or gene silencing approaches are needed to establish a causal link between JNK pathway suppression and the wound-healing effects of EV-exoLF. Future research will aim to clarify this relationship through functional assays that dissect the molecular contributions of JNK and other pathways in fibroblast-driven wound repair.

In recent years, exosomes derived from mesenchymal stem cells (MSCs) and various synthetic wound-healing agents have been extensively investigated for their regenerative and anti-inflammatory properties [82]. Although our study did not include a direct comparison, the biological activity of colostrum-derived EV-exoLF observed in this study—namely its ability to reduce inflammatory cytokine levels, suppress p-JNK expression, and enhance fibroblast migration—suggests that it may serve as a promising alternative or complementary approach. Compared to MSC-derived exosomes, colostrum-derived EVs offer several practical advantages, including non-invasive and ethically uncomplicated sourcing, cost-effective scalability, and a naturally rich cargo of immunomodulatory proteins and growth factors such as lactoferrin. These components may work synergistically to promote wound healing and tissue repair. While synthetic agents can accelerate wound closure, they may lack the bioactive complexity and biocompatibility inherent to naturally derived exosomes. Future studies will be necessary to perform head-to-head comparisons between EV-exoLF, MSC-derived exosomes, and conventional wound-healing formulations in standardized in vitro and in vivo models to validate relative efficacy and safety.

NIH/3T3 and HS-68 cell lines were utilized in this study to evaluate the cytocompatibility, inflammatory response, and wound-healing potential of EV-exoLF. While these cell lines are commonly used and offer reproducible, well-characterized models for studying basic fibroblast functions such as migration, cytokine response, and matrix remodeling [83], they do not fully replicate the complexity of in vivo wound healing, which involves dynamic interactions among multiple cell types, extracellular matrix components, vascularization, and immune responses. Specifically, HS-68 human dermal fibroblasts were selected for their relevance to human tissue physiology, particularly in the context of inflammation and dermal repair [84]. Nonetheless, we recognize the limitations of using 2D monolayer cultures and acknowledge that further validation in 3D tissue-engineered skin models or animal wound healing models will be essential to confirm the translational relevance of our findings.

The observed anti-inflammatory and wound-healing effects of EV-exoLF may be attributed not only to the biological activity of lactoferrin but also to the exosomal delivery mechanism itself. Exosomes act as natural nanocarriers that enhance the stability, bioavailability, and cellular uptake of their cargo [85]. This is particularly important for protein-based therapeutics such as lactoferrin, which are otherwise susceptible to rapid degradation in biological environments [86]. Although lactoferrin is known to exhibit potent anti-inflammatory and immunoregulatory properties, the encapsulation of lactoferrin within exosomes (EV-exoLF) may facilitate more efficient intracellular delivery and targeted effects. While our study did not include free LF control, prior studies have indicated that exosomal formulations often outperform free compounds due to enhanced delivery kinetics. Future experiments will include a direct comparison of EV-exoLF versus free lactoferrin to better define the independent contribution of the exosomal carrier system to the therapeutic effects observed.

While this study focused on the functional outcomes of EV-exoLF treatment in fibroblast cells, the precise mechanism by which EV-exoLF is internalized was not directly examined. Exosomes are known to be internalized by various cell types through mechanisms such as clathrin-dependent endocytosis, caveolin-mediated uptake, macropinocytosis, and receptor–ligand interactions, with the exact pathway depending on both the exosome surface proteins and the recipient cell type [87]. Although it is reasonable to assume that EV-exoLF enters fibroblasts via similar endocytic routes, direct evidence such as exosome tracking with fluorescent dyes or inhibition assays using pathway blockers was not collected in this study. Future experiments will aim to elucidate the uptake mechanism of EV-exoLF in fibroblasts, which will provide a better understanding of its intracellular trafficking and the efficiency of bioactive cargo delivery.

Although this study demonstrated the anti-inflammatory and pro-migratory effects of EV-exoLF in fibroblast cells, further experiments are warranted to comprehensively evaluate its wound-healing potential. In particular, cell proliferation assays and the assessment of key pro-regenerative and angiogenic factors, such as transforming growth factor-beta (TGF-β), vascular endothelial growth factor (VEGF), endothelial nitric oxide synthase (eNOS), and collagen I, would offer valuable insights into the molecular pathways involved in tissue remodeling and repair [88,89,90,91]. These markers could be assessed at the transcriptional level via RT-PCR or at the protein level using immunofluorescence staining, following EV-exoLF treatment. While these experiments were not included in the current study due to technical constraints, future work will incorporate these assays to better define the multifaceted biological effects of EV-exoLF in wound healing and skin regeneration.

This study provides foundational in vitro evidence supporting the cytocompatibility, anti-inflammatory effects, and wound-healing potential of EV-exoLF in fibroblast cultures. However, we acknowledge that the absence of in vivo validation represents a key limitation. Animal models are crucial for assessing the complex physiological processes of wound healing, including vascularization, immune modulation, tissue remodeling, and systemic biodistribution of the therapeutic agent. While our findings suggest promising therapeutic properties of EV-exoLF, further animal studies such as excisional or burn wound models in rodents are necessary to evaluate its efficacy and safety in a biologically integrated environment. These future investigations will help determine the clinical relevance and translational potential of EV-exoLF in regenerative medicine.

## 5. Conclusions

This study demonstrated the therapeutic potential of colostrum-derived EV-exoLFs in promoting the self-healing capacity of skin fibroblasts by modulating inflammatory responses. Overall, our findings suggest that EV-exoLFs mitigate inflammatory damage and support tissue regeneration in dermal fibroblasts. The natural origin, nanoscale vesicle structure, and immunomodulatory function of EV-exoLF position it as a promising bioactive candidate for the future development of anti-inflammatory and wound-healing therapeutics. However, further in vivo studies are warranted to validate its clinical efficacy and pharmacological safety.

In conclusion, our results showed that the in vitro milk-derived EV-exoLF exhibited tissue remodeling activity, resulting in enhanced tissue repair and regeneration. Thus, ExoLF may play modulatory and anti-inflammatory roles in immune responses. Based on our findings, EV-exoLF may have potential topical therapeutic applications in the treatment of facial wounds. Ultimately, despite the protective nature of EV-exoLF, further in vivo and in vitro studies are warranted to explore interactions between cells and the inflammatory microenvironment.

## Data Availability

The data supporting the findings of this study are available from the corresponding author upon reasonable request.

## Abbreviations

The following abbreviations are used in this manuscript:

α-SMA	α-Smooth muscle actin
ADSC	Adipose-derived mesenchymal stem cell
Col1	Collagen I
Col3	Collagen III
DMEM	Dulbecco’s Modified Eagle’s Medium
ECM	Extracellular matrix
ELISA	Enzyme-linked immunosorbent assay
EV	Extracellular vesicle
EV-exoLF	Exosome-encapsulated lactoferrin
HRP	Horseradish peroxidase
IL	Interleukin
LF	Lactoferrin
MSC	Mesenchymal stem cell
PBS	Phosphate-buffered saline
pJNK	Phosphorylated c-Jun N-terminal kinase
